# Long-term outcome of sacral neuromodulation for chronic refractory constipation

**DOI:** 10.1007/s10151-017-1613-0

**Published:** 2017-04-20

**Authors:** Yasuko Maeda, Michael A. Kamm, Carolynne J. Vaizey, Klaus E. Matzel, Claes Johansson, Harald Rosen, Cornelius G. Baeten, Søren Laurberg

**Affiliations:** 1 Sir Alan Parks Physiology Unit, St. Mark’s Hospital, Harrow, UK; 2 Imperial College, London, UK; 3 Department of Gastroenterology, St. Vincent Hospital Melbourne, Melbourne, Australia; 4 University of Melbourne, Melbourne, Australia; 5 Chirurgische Klinik mit Poliklinik der Friedrich Alexander, Universität Erlangen/Nürnberg, Erlangen, Germany; 6 Department of Surgery, Danderyd Hospital, Danderyd, Sweden; 7 Vienna Private Clinic, Vienna, Austria; 8 Department of Surgery, Maastricht University Medical Centre, Maastricht, The Netherlands; 9 Department of Surgery P, Aarhus University Hospital, Aarhus, Denmark

**Keywords:** Sacral neuromodulation, Sacral nerve stimulation, Constipation

## Abstract

**Purpose:**

Sacral neuromodulation has been reported as a treatment for severe idiopathic constipation. This study aimed to evaluate the long-term effects of sacral neuromodulation by following patients who participated in a prospective, open-label, multicentre study up to 5 years.

**Methods:**

Patients were followed up at 1, 3, 6, 12, 24, 36, 48 and 60 months. Symptoms and quality of life were assessed using bowel diary, the Cleveland Clinic constipation score and the Short Form-36 quality-of-life scale.

**Results:**

Sixty-two patients (7 male, median age 40 years) underwent test stimulation, and 45 proceeded to permanent implantation. Twenty-seven patients exited the study (7 withdrawn consent, 7 loss of efficacy, 6 site-specific reasons, 4 withdrew other reasons, 2 lost to follow-up, 1 prior to follow-up). Eighteen patients (29%) attended 60-month follow-up. In 10 patients who submitted bowel diary, their improvement of symptoms was sustained: the number of defecations per week (4.1 ± 3.7 vs 8.1 ± 3.4, mean ± standard deviation, *p* < 0.001, baseline vs 60 months) and sensation of incomplete emptying (0.8 ± 0.3 vs 0.2 ± 0.1, *p* = 0.002). In 14 patients (23%) with Cleveland Clinic constipation score, improvement was sustained at 60 months [17.9 ± 4.4 (baseline) to 10.4 ± 4.1, *p* < 0.001]. Some 103 device-related adverse events were reported in 27 (61%).

**Conclusion:**

Benefit from sacral neuromodulation in the long-term was observed in a small minority of patients with intractable constipation. The results should be interpreted with caution given the high dropout and complication rate during the follow-up period.

## Background

Constipation is one of the common bowel disorders seen in daily clinical practice. The prevalence is estimated to be around 14% with significant impact on quality of life, in some patients to debilitating effect [1].

The initial management of constipation is lifestyle advice such as sufficient fluid and fibre intake although the evidence is poor. Use of laxatives is common but often not judicious and many patients remain refractory to currently available laxatives. Prucalopride and other recently available prokinetic drugs are effective for some patients in short term [2], but their efficacy may not be sustained in the long term. Behavioural treatment (biofeedback) is effective for many patients in a randomized trial, but some remain symptomatic [3, 4].

Bowel irrigation can be utilized either retrograde (via anus) or antegrade via creation of irrigation stoma (appendicostomy or caecostomy) which in some patients exacerbates sensation of bloating and abdominal pain. Traditional surgical approach has been colectomy with stoma formation which is associated with complications and necessitates life-long use of stoma bags and should only be considered once all other options have been exhausted [5].

Sacral neuromodulation (SNM) is a minimally invasive treatment which has become an established option for faecal incontinence over the last decade in Europe. Ganio et al. [6] first reported improvement of constipation in patients treated by SNM. Since then a few other studies emerged that SNM may be efficacious for constipation refractory to conventional treatment [7–11]. However, the follow-ups have been in general short- or medium-term and there is a paucity of knowledge of treatment outcome in the long term.

This study aimed to evaluate the outcome of SNM for constipation refractory to medical and behavioural treatment over the 5-year study period.

## Methods

Data have been prospectively collected in an open-label, multicentre study to evaluate the clinical efficacy and safety of sacral neuromodulation for chronic intractable constipation. Data were recorded up to 60-month follow-up. Study methods, including baseline investigations, the definition of constipation, inclusion and exclusion criteria, and operative details have been reported previously [11]. Briefly, data collected at baseline included patient demographics, bowel diary, patient’s rating of his/her bowel habit on a visual analogue scale (VAS), Cleveland Clinic constipation score (CCCS, Wexner Constipation Score) [12], 36-item Short Form Health Survey (SF-36) [13] and use of medication. Inclusion criteria were as follows: (a) age between 18 and 75 years, (b) chronic constipation documented by baseline diary 3 weeks as defined by two or fewer bowel movements per week on average and/or impaired defecation defined as >25% of all visits to the bathroom (attempts to defecate) subject had to strain and/or >25% of all visits to the bathroom subject did not feel empty afterwards (incomplete evacuation), (c) symptoms of constipation present for a minimum of 1 year, (d) failed maximal medical therapy such as dietary modifications, laxatives and enemas, (e) failed biofeedback therapy within 1 year before enrolment. Exclusion criteria were (a) any organic pathology that may be causing constipation and requiring surgical intervention, (b) congenital anorectal malformations, (c) previous large bowel surgery including rectal prolapse repair, (d) present rectal prolapse, (e) chronic bowel diseases such as inflammatory bowel disease, (f) inconsistent bowel habit, associated with alternating constipation and diarrhoea, (g) stoma in situ, (h) neurological diseases, such as complete spinal cord transection, multiple sclerosis, spina bifida and Parkinson’s disease, (i) subjects who have a significant psychological and social element to their symptoms as judged by the investigator, (j) bleeding complications, (k) pregnancy, (l) anatomical limitations which would prevent successful placement of an electrode, (m) skin and tissue diseases with the risk of infection such as pyoderma and untreated pilonidal sinus.

All patients underwent a 3-week test stimulation with the InterStim model 3057 lead prior to permanent device implantation. It should be noted that the CE mark approved duration for test stimulation with the model 3057 lead is 7 days. For the purpose of this trial, the lead was specifically marked as investigational device allowing up to 30 day of test period. Competent authorities were informed. Criteria to proceed to permanent implantations were as follows: (1) weekly average of bowel movements improved to ≥3 without increase of laxatives, enemas or manual stimulation, and/or (2) ≥50% of the number of episodes with impaired defecation (straining or incomplete evacuation depending on the clinical situation at baseline).

After permanent device implantation, patients were followed up at 1, 3, 6, 12, 24, 36, 48 and 60 months using the same set of questionnaires. Anorectal physiological testing was performed at each follow-up. Programming changes were done as per standard clinical practice as and when needed to optimize the therapy. Colonic transit study and evacuation proctogram were repeated at 6 months post-implant. Therapeutic efficacy of permanent SNM was evaluated by comparing baseline with post-implant data obtained at follow-up visits.

### Adverse events

All adverse events during the study were documented, regardless of whether they were related to the treatment. Events were classified as serious and non-serious adverse events or an adverse device effect according to ISO 14155. Each event was further rated as mild, moderate or severe.

### Statistical analysis

The principle of intention to treat (ITT) was used to analyse the data for outcome. This meant that all participants who underwent PNE in the study were included in the analysis. The proportion of patients at each follow-up is expressed in both ITT and per protocol analysis (PP) that included only those subjects who underwent an implantation of device. This approach was used to measure the effects of SNM on constipation and to reflect what would happen in clinical practice.

Data are presented as mean and standard deviation when normally distributed and median and range when not normally distributed. When data distribution changed over follow-up period, both sets of data are presented. Quantitative or continuous data are summarized by descriptive statistics as number of values, while qualitative or discrete data are summarized as frequency and percentage of patients in each class variable.

All analyses of the efficacy variables were performed by fitting a generalized estimating equations (GEE) model for repeated data based on a normal distribution. The model had the efficacy variables as dependent variables and baseline values and visits as explanatory variables.

The efficacy variables could have been pre-log transformed depending on their normal or non-normal distribution. The visits explanatory variables were considered as a qualitative variable with baseline visit as the reference level. SAS PROC GENMOD was used to conduct the GEE analysis using an unstructured correlation structure. Difference between visits means was tested by applying contrast on the estimated GEE model.

Statistical tests were two-sided, and a p value of less than 0.05 was considered statistically significant. Since this was an exploratory study, no imputation was implemented for missing follow-up data. The bowel diary did not differentiate a day without defecation from a day with missing data. Bowel diary data were handled as follows: (1) monitored days were defined as days for which there was at least one entry for any of the variables, (2) in case no data were collected for one or more days in the diary, these days were considered as missing days and no imputation was implied, (3) in case data from the diary were missing for a whole visit, no imputation was implemented and the diary was considered missing.

For the CCCS questionnaire, imputation was performed when data were not available for all items of the questionnaire as follows: (1) when one or several items of the Wexner score were missing for a completed visit, imputation of data using the last observation carried forward (LOF) approach was used (the last observation had to originate from a post-baseline visit), (2) when the entire CCCS score was missing for a completed visit, no imputation was implemented and the score value considered as missing.

No imputation was applied for missing data from the SF-36 questionnaire, as SF-36 scores can be computed from incomplete SF-36 questionnaires through standard SF-36 algorithms.

In case a subject terminated the study early, no imputation was implemented for the missing visits.

## Results

The study was started in January 2002 and completed in December 2012. Sixty-two (62) patients (7 male, median age of 40, range 17–79) at 5 European centres [St Mark’s Hospital, London, UK (30 patients); Maastricht University Medical Centre, Maastricht (17), The Netherlands; Aarhus University Hospital, Aarhus, Denmark (8); Danderyd Hospital, Stockholm, Sweden (4); and Danube Hospital, Vienne, Austria (3)] were enrolled in the study and underwent test stimulation. Symptoms of constipation had been present for a median duration of 10 (range 1–60) years prior to enrolment. Fifty patients (81%) had slow transit constipation, and 12 (19%) had normal colonic transit.

Forty-four (71%) patients fulfilled the criteria for permanent implant, while 18 patients (29%) did not fulfil the criteria. Of these 44, one patient withdrew from the study before implant. Two other patients underwent permanent implantation despite not fulfilling the study implant criteria. These patients were included by errors, and this was only recognized after data audit. One had previous rectal prolapse surgery and the second patient had an underlying neurological disease; hence, these were regarded as protocol deviation from exclusion criteria. Forty-five patients underwent permanent implantation, of whom 44 attended at least one follow-up. One patient experienced leg pain after permanent implantation and exited the study before the 1-month follow-up.

Twenty patients (32% of tested patients, 45% of implanted patients) who had a device implanted or exited the study prematurely. Seven patients withdrew consent from the study. Seven patients exited due to loss of efficacy. The mean interval between implantation and loss of efficacy was 30.3 ± 15.1 months. Six patients were not available for 60-month follow-up for a site-specific reason.

**Fig. 1:**
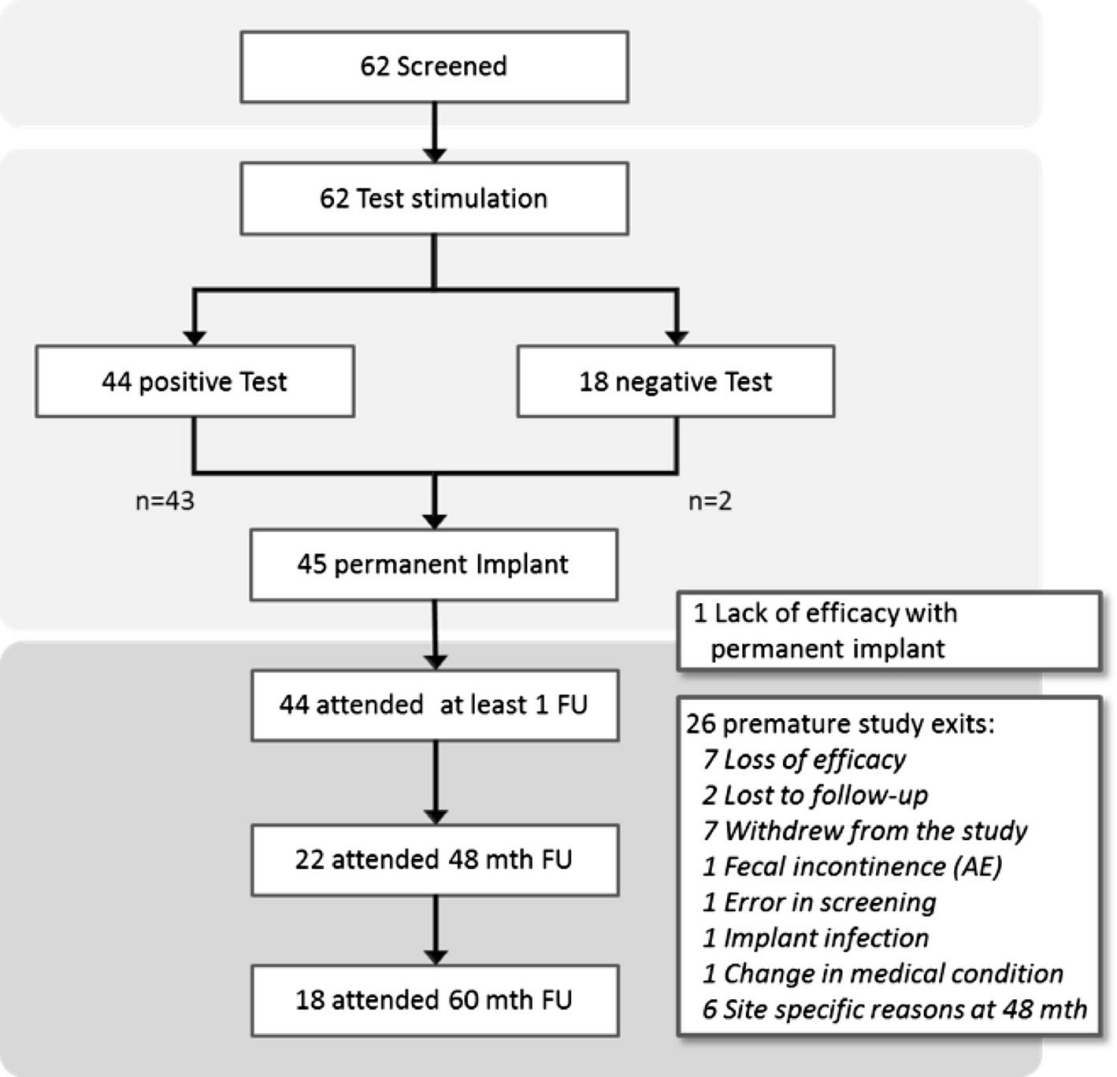
Flow of patients

Two patients were lost to follow-up. One patient exited for each of the following reasons: the patient did not fulfil the inclusion criteria and entered by error, change in medical condition, faecal incontinence and infection around the implanted device. Eighteen patients attended 60-month follow-up. The flow of study patients is summarized in Fig. 1.

### Bowel diary data

**Fig. 2:**
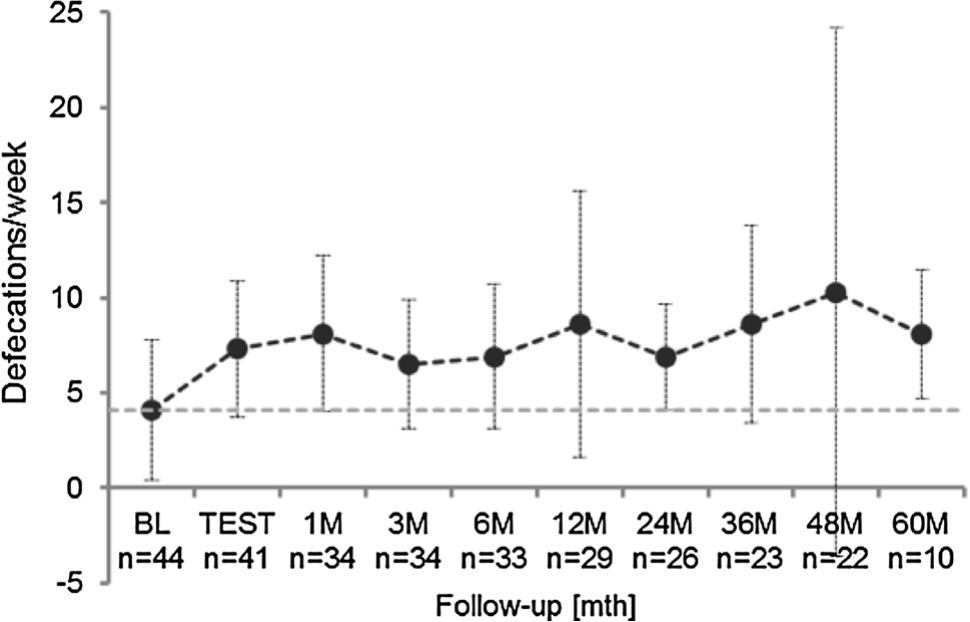
Mean number of defecations per week (mean ± SD) at baseline (BL), test stimulation and up to 60 months after implant. (*n* = number of patients at each follow-up)

The effects of chronic SNM on constipation symptoms at follow-up were assessed using the bowel diary. The number of bowel diaries completed at baseline and test stimulation was 44 out of 62 patients (ITT: 71%), at 48 months 21 out of 22 patients (ITT: 34%, PP: 48%) and 10 out of 18 patients (ITT: 16%, PP: 23%) at 60-month follow-up. The number of defecations per week increased from 3.3 (0.0–20.3, 4.1 ± 3.7, *N* = 44) [median (range, mean ± standard deviation, number of valid diaries)] at baseline to 6.7 (0.6–17.2, 7.3 ± 3.6, *N* = 41) at test stimulation, 7.0 (1.0–70.0, 10.3 ± 13.9, *N* = 22) at 48 months and 7.2 (3.0–15.7, 8.1 ± 3.4, *N* = 10) at 60 months (*p* < 0.001) (Fig. 2). The number of days per week with successful defecation increased from 2.3 (0.0–7.0, 2.8 ± 2.0, *N* = 44) at baseline to 5.0 (0.6–7.0, 4.9 ± 1.7, *N* = 41) during the test stimulation, 4.7 (1.0–7.0, 4.7 ± 2.0, *N* = 22) at the 48-month visit and 5.4 (2.7–7.0, 5.2 ± 1.5, *N* = 10) at 60 months (*p* < 0.001). There were statistically significant improvement of proportion of successful evacuations, the average proportion of successful evacuations associated with a sensation of incomplete emptying, time spent on toileting per defecation, the number of days with no abdominal pain or discomfort, and the number of days with no abdominal bloating all improved at 60 M compared to baseline. The proportion of spontaneous bowel movements increased significantly from 0.4 (0.0–1.0, 0.5 ± 0.4) at baseline to 1.0 (0.0–1.0, 0.9 ± 0.2) during the test stimulation and remained significant for all follow-up visits (*p* < 0.005), except at 1, 12 and 36 months.

Patient subjective assessment of bowel habit was rated 0.08 (0.0–1.0, 0.15 ± 0.22) out of 1 on average at baseline (0: very poor, 1: very good). The rating improved to 0.77 (0.0–1.0, 0.74 ± 0.24) during the test stimulation, 0.62 (0.01–1.0, 0.55 ± 0.34) at 48 months and 0.85 (0.59–0.93, 0.82 ± 0.1) at 60 months (*p* < 0.001). The details of bowel diary are summarized in Table 1.

**Table 1:** Bowel diary data at baseline and follow-up

	Baseline (*n* = 44)	TEST (*n* = 41)	1 M FU (*n* = 34)	3 M FU (*n* = 34)	6 M FU (*n* = 33)	12 M FU (*n* = 29)	24 M FU (*n* = 26)	36 M FU (*n* = 23)	48 M FU (*n* = 22)	60 M FU (*n* = 10)
Frequency of defecation (number of defecations/week)	4.1 (3.7)	7.3 (3.6)	8.1 (4.1)	6.5 (3.4)	6.9 (3.8)	8.6 (7)	6.9 (2.8)	8.6 (5.2)	10.3 (13.9)**	8.1 (3.4)
Days per week with successful defecation per week	2.8 (2.0)	4.9 (1.7)	5.2 (1.6)	4.6 (1.9)	4.7 (1.9)	5.2 (1.7)	5 (1.7)	5.3 (1.9)	4.7 (2.0)	5.2 (1.5)
Use of laxative, suppository or enema (number of days/week)										
Laxative	1.0 (1.8)	0.6 (2.2)	0.7 (1.9)	0.5 (1.2)	1 (2)	1.9 (3.7)	1.3 (2.7)	1.8 (3.7)	2 (4.2)	4.3 (5.4)
Enema	0.8 (1.7)	<0.05 (0.1)	0.2 (0.6)	0.3 (1.0)	0.1 (0.2)	0.1 (0.3)	0.1 (0.2)	0.1 (0.3)	0.1 (0.5)	<0.05 (<0.05)
Suppository	0.1 (0.4)	<0.05 (0.1)	0.0 (0.2)	0.1 (0.2)	0.1 (0.3)	<0.05 (0.1)	<0.05 (0.1)	0.2 (0.8)	0.1 (0.4)	<0.05 (<0.05)
Finger used	0.4 (1.3)	0.3 (1.1)	0.2 (0.6)	0.3 (0.8)	0.4 (1.2)	0.4 (1.3)	0.2 (0.6)	0.4 (1)	0.6 (1.4)	0.3 (0.7)
Proportion of successful defecations that required patient to strain	0.8 (0.3)	0.4 (0.3)	0.4 (0.3)	0.4 (0.3)	0.5 (0.3)	0.5 (0.3)	0.5 (0.4)*	0.4 (0.3)*	0.5 (0.4)*	0.6 (0.4)^
Proportion of successful defecations associated with a sensation of incomplete emptying	0.8 (0.3)	0.4 (0.3)	0.4 (0.3)	0.5 (0.4)	0.4 (0.3)	0.4 (0.3)	0.4 (0.3)^+^	0.4 (0.3)^+^	0.5 (0.3)^+^	0.2 (0.1)**
Time spent on toilet per defecation [min]	16.7 (15.4)	8.3 (6.1)*	7.8 (6)	9.9 (8.7)*	8.1 (5.2)	8.1 (6.7)**	7.4 (5.9)**	10 (14.1)^†^	10.8 (18)**	8.3 (5.8)^†^
Proportion of spontaneous bowel movements/week	0.5 (0.4)	0.9 (0.2)**	0.8 (0.3)^¶^	0.8 (0.4)^†^	0.8 (0.4)^§^	0.7 (0.4)^¶^	0.7 (0.4)	0.6 (0.4)^¶^	0.8 (0.4)°	0.7 (0.4)^
Limitation in daily activities resulting from constipation [number of days/week]	2.3 (2.3)	0.8 (1.4)	0.8 (1.7)	1 (1.8)	0.8 (1.6)	0.7 (1.4)	0.6 (1.1)	1.2 (2.0)	1.3 (2.3)	0.4 (1.3)
Number of days without abdominal bloating per week	1.3 (2.0)	3.5 (2.3)**	3.4 (2.6)^†^	3.2 (2.8)*	3.9 (2.7)*	3.6 (2.6)*	3.4 (2.7)^#^	3.8 (2.5)^§^	3.1 (2.3)^¶^	
Number of days with no pain	1.4 (2.0)	4.2 (2.2)	4.3 (2.5)	3.9 (2.8)	4 (2.3)	3.6 (2.4)	4.2 (2.5)	3.7 (2.5)	3.7 (2.6)	

All values are expressed as mean (SD). All values changed significantly as compared to baseline with *p* < 0.001 if not indicated as * *p* = 0.001, ** *p* = 0.002, ^+^ *p* = 0.003, ^†^ *p* = 0.004, ^#^ *p* = 0.005, ^§^ *p* = 0.01, ^ *p* = 0.02, ° *p* < 0.05, ^¶^  not significant

### The Cleveland Clinic constipation score

**Fig. 3:**
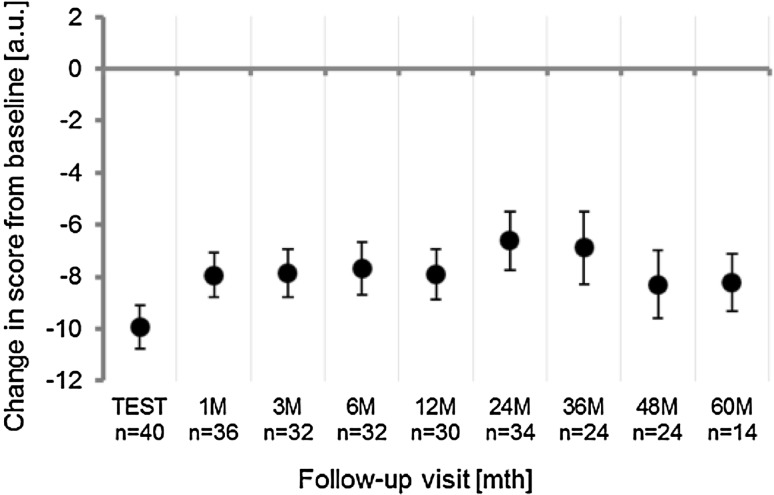
Change in CCCS score from baseline (mean ± SD). A negative change indicates decreased severity

Fourteen patients (ITT: 23%, PP: 31%) had both the baseline and at 60 months CCCS. The Cleveland Clinic constipation score (0 = no symptoms of constipation to 30 = severe constipation) was 18 (11–27, 17.9 ± 4.4, *N* = 42) (median (range, mean ± standard deviation)) at baseline and 7 (2–19, 8.4 ± 4.2, *N* = 42) during test stimulation. The median score was 11 (3–21, mean 11.2 ± 5.1, *N* = 26) at 48 and 12 (3–17, mean 10.4 ± 4.1, *N* = 16) at 60 months. Scores across the follow-up period were significantly reduced compared to baseline with *p* value of less than 0.001. Scores over the follow-up period are summarized in Fig. 3.

### Quality of life

**Fig. 4:**
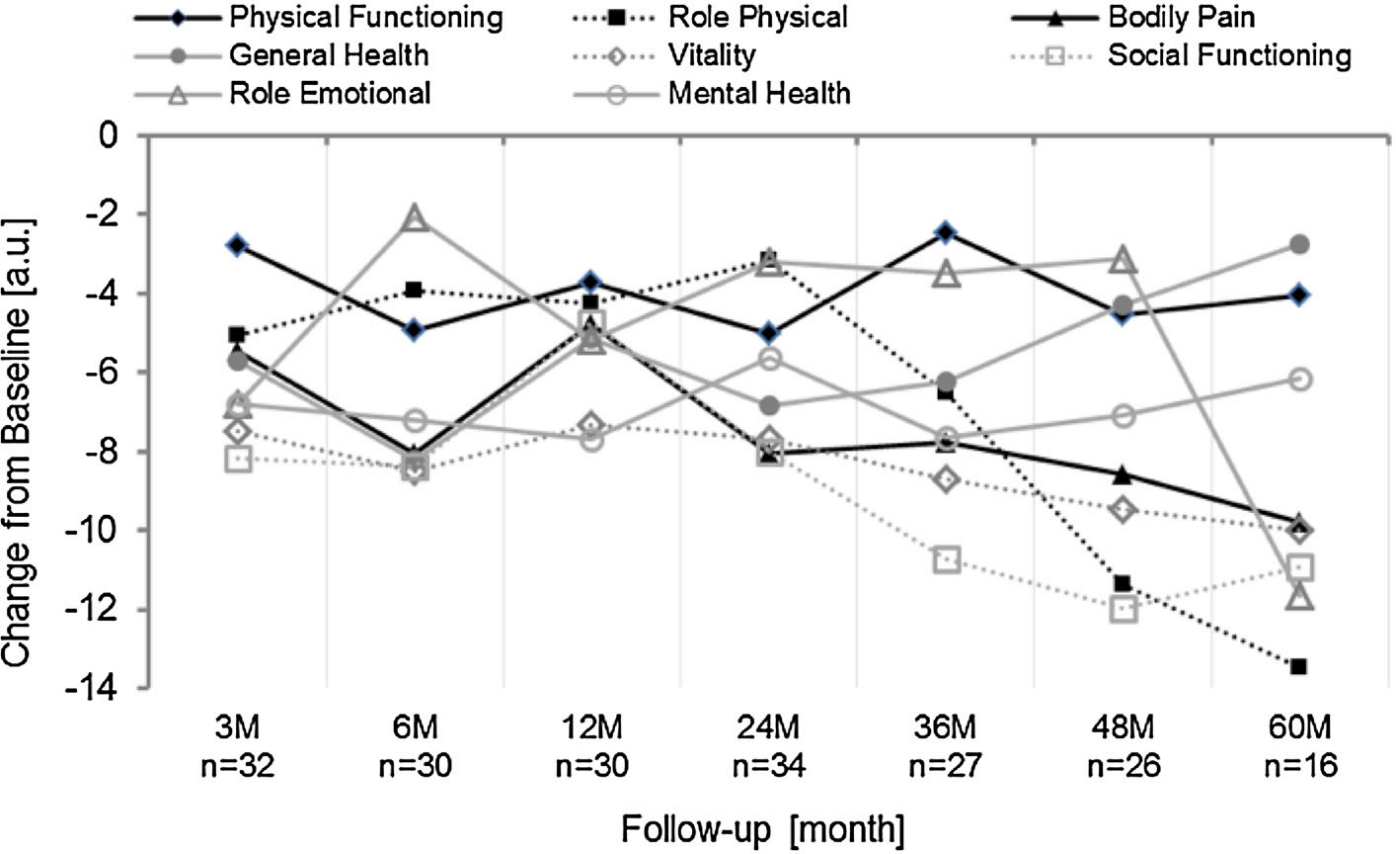
Change in HRQoL measured with the SF-36 from baseline

SF-36 questionnaires were available from 38 out of 62 (ITT: 61%) at baseline, 22 out of 22 patients (ITT: 35%, PP: 50%) at 48 months and 16 out of 18 patients (ITT: 26%, PP: 36%) at 60 months. Quality of life was not assessed at test stimulation. All SF-36 scores and composite scores improved during test stimulation and after permanent implant as compared to baseline. The improvement was maintained over the follow-up period, but did not reach statistical significance at all follow-up visits. At 48 and 60 months, the improvement was statistically significant as compared to baseline in the following domains: physical functioning (*p* < 0.02), role physical (*p* < 0.04), bodily pain (*p* < 0.001), vitality (*p* < 0.001), social functioning (*p* < 0.001) and mental health (*p* < 0.012) as well as for the physical and mental composite scores. Improvement at emotional role was significant at 60 months (*p* < 0.01). There was no statistically significant change in the general health at 48 and 60 months and emotional role at 48 months (*p* > 0.1) (Fig. 4).

### Anorectal physiological data

**Fig. 5:**
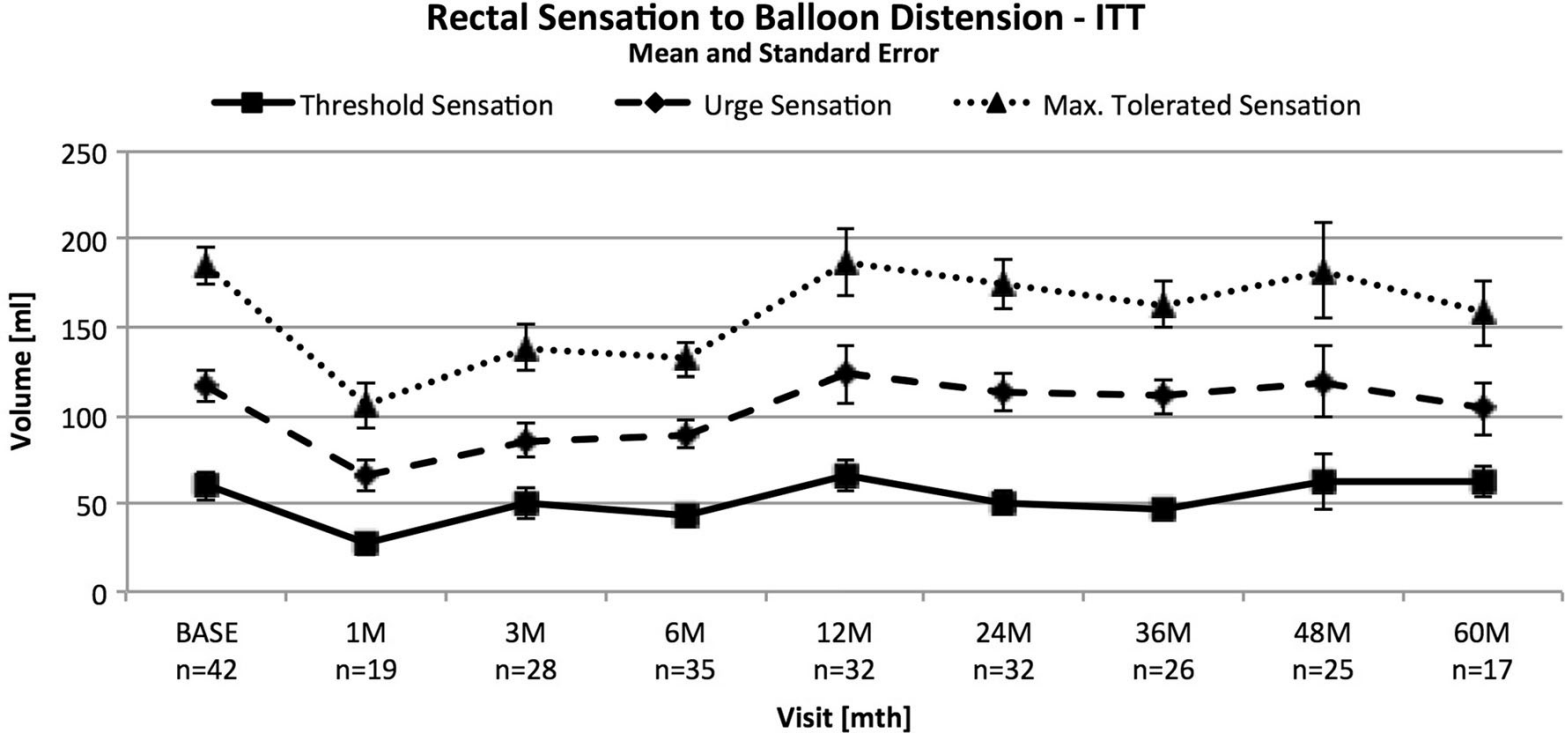
Change in rectal volumes from baseline to follow-up under SNM

The maximal mean resting pressure, maximal mean squeeze pressure and length of the high pressure zone did not show a significant difference between baseline, test stimulation and follow-up assessments. The rectal volumes for urge, threshold of sensation and maximal tolerated sensation decreased from baseline for the 1–6-month visits, but this decrease was not maintained at mid- and long-term follow-ups (Fig. 5).

### Colonic transit study

Thirty-six patients out of 44 who fulfilled the criteria for permanent implantation had colonic transit time study done at baseline. Of these patients, 9 patients did not have colonic transit time at 6 months, leaving only 27 patients available for comparison between baseline and at 6 months: twenty patients had slow transit, and 7 patients had normal transit. Of 20 patients with slow transit, 12 patients had normal transit at 6 months, while 8 had persistent slow transit time. Of 7 patients with normal transit, 4 patients remained normal and 3 patients had slow transit at 6 months.

Of 18 patients who were available for follow-up at 60 months, 13 patients had colonic transit time done both at baseline and at 6 months. Eleven patients had changed from slow to normal, 1 patient was normal at baseline and slow at 6 months, and 1 patient remained slow. A statistical analysis to evaluate whether the change of transit time change from baseline to 6 months had any impact on the outcome at 60 months was attempted but not possible due to small sample size.

### Subgroup analysis

We have conducted a separate analysis of those patients who attended 60 months follow-up, comparing their baseline and 60-month data. All the parameters such as the number of defecations per week (*p* = 0.016), the number of days per week with successful defecation (*p* = 0.02), proportion of successful evacuations with sensation of incomplete emptying (*p* = 0.08), VAS score (*p* = 0.016) and Cleveland Clinic constipation score (*p* = 0.01) and physical functioning (*p* = 0.02) of SF36 were improved. The only subanalysis which altered from the GEE analysis was the number of natural bowel movements which was non-significant in the subanalysis (*p* = 0.156).

### Adverse events

A total of 216 adverse events occurred in 41 (66%) of tested patients: thirteen of them occurred during testing phase and 203 events in 39 of 45 implanted patients (87%). Sixty-four (64) adverse events in 22 (35%) of tested patients were classified as serious: six of them occurred before the definitive IPG implantation and 58 after implantation in 20 of the 45 implanted patients (44%).

The most frequently recorded adverse event (60 events) was ‘gastrointestinal disorders’ including abdominal pain (15 events) and constipation (11 events) in 21 patients (34% of tested patients, 47% of implanted patients). The second commonest adverse event was classified as ‘general disorders and administration site conditions’ including device-related events including computer issues, device dislocation, lead damage or implant site pain (total 47 events in 23 patients (37% of tested patients, 49% of implanted patients). This was followed by events categorized as ‘musculoskeletal and connective tissue disorders’ including limb pain/discomfort and musculoskeletal pain and spasm (36 events) that occurred in 20 patients (32% of tested patients, 42% of implanted patients).

A total of 122 adverse events were related to the device: nineteen of them occurred during of after the test period, and 103 events occurred in 27 (61%) patients after implantation. Twenty-three (23) events were serious adverse device effects and were observed in 12 (19%) of the tested patients, including 17 which occurred after device implantation in 10 patients (22% of implanted patients). Serious adverse device effects accounted for 10% of all adverse events. All adverse device effects and serious adverse device effects were anticipated adverse events. One hundred and four events (46% of total events) occurring in 46 (74%) of tested patients were classified as not being related to the use of the device.

Four adverse events led to 4 patients (% of implanted patients) withdrawal from the study. Three (3) of those were adverse device effects after implantation, and one patient had infection at connection between the tined lead and connector to extension lead which eventually led to withdrawal post-implantation.

Women who were pregnant or considering getting pregnant were excluded from study participation. However, 3 patients had a total of 5 pregnancies during the course of the study. Two of those in one patient have been described previously [11]. One other patient also had two pregnancies. She informed the investigator of her intention to become pregnant, and the stimulation was turned off prior to conception for both pregnancies. Two healthy babies were born at term. In case of the third pregnancy, the stimulation was turned off at the 5th week of gestation. The healthy baby was delivered at term. In all pregnancies, delivery was by caesarian section. At the time of last follow-up, there was no unresolved adverse event.

### System modifications

System modification was required on 21 occasions in 14 (31%) patients. Revisions of one of the implanted components (neurostimulator, lead, extension) were required on 5 occasions in 4 (9%) patients, replacement on 11 occasions in 10 (22%) patients, and removal of one of the components on 5 occasions in 3 (7%) patients. Reasons for system modification were specified for 10 patients as lead migration on 4 occasions in 4 (9%) patients, infection on 2 occasions in 2 (4%) patients, suspected device problem on 1 occasion in 1 (2%) patient, and other on 12 (63%) occasions in 10 (22%) patients.

### Other data

The number of laxatives used was 1.0 (0.0–8.7, 1.0 ± 1.8, *N* = 44) [mean (range, mean ± standard deviation, number of valid diaries)] at baseline which increased to 2.0 (0.0–15.7, 2.0 ± 4.2, *N* = 22, ITT: 35%, PP: 49%) at 48 M and 4.3 (0.0–14.3, 4.3 ± 5.4, *N* = 10, ITT: 16%, PP: 22%) at 60 M.

## Discussion

Constipation is a relatively new indication of sacral neuromodulation. The current gap in the treatment options for this condition left clinicians in search of an approach that would not render patients dependent on chronic laxative use while eliminating the need for major surgery. Initial promising results of SNM for constipation [14] led to this prospective trial.

Thirty-five and 18 of the patients who had been considered for this treatment were available at 48 and 60 months, and the bowel diary was completed by 22 of 35, and 10 of 18 patients, respectively. Just under 50% of patients were available for 48-month follow-up which was further reduced at 60 months partly due to one of the study sites missing the follow-up window despite that the patients were still using SNM therapy. This is a small proportion of the original patient cohort. This illustrates one of the many challenges of multicentre, long-term study. Filling in a detailed bowel diary over a long study period at various time points was a practical challenge, and we acknowledge that there may be both attrition bias and reporting bias whereby only those patients who had good outcome from the therapy filled in bowel diary. As the study used per protocol analysis by following up only those patients with the SNM device on and did not include patients who exited study, it could be argued that there is a probable bias for the results to be in positive direction given that the study was also uncontrolled and the patients knew they were on active treatment. However, analysis of those who exited the trial was not possible due to regulatory binding that we are obliged to report this trial outcome as per protocol.

It is also not possible to ascertain whether patients who benefitted long term did so because of the SNM or other factors. Laxative use at 5 years was similar to baseline. Data on dose were only reported anecdotally so that no comparison on the amount of laxatives used could be done. Nevertheless, the continued use of laxatives at 48 and 60 months suggests that although symptoms were improved on long-term follow-up, there was still a need for supplementary therapy.

The mechanisms of action of SNM for constipation remain obscure. A recently published study [15] postulated that SNM may normalize rectal hyposensitivity in patients with evacuatory dysfunction. Our findings showed a reduction of maximum volume at 3 months, but this effect was lost afterwards. Rectal sensory volumes were similar at 60 months to baseline. Suprasensory stimulation (a stimulation above sensory perception threshold) has been shown to increase colonic propagation which suggests the SNM affects afferent pathway modulation [16] although the precise mechanism is unclear. A recent randomized controlled trial by the same authors, however, showed no difference in the number of complete bowel movements between sham, subsensory and suprasensory stimulation [17]. Although it is likely that SNM modulates both local and central pathways, data to date are too sparse to allow a meaningful understanding of the association between the physiological findings and improvement of clinical symptoms.

A recent study showed the underlying type of constipation does not influence the outcome of SNM [18]. The current study did not allow us to determine whether those with predominantly slow transit or evacuation difficulties benefited more from SNM. This is because the number of patients who were available at 60 months was small. There were also a few patients who continued this therapy beyond 60 months but were treated as exited the study due to failure to follow-up at the trial window, and thus, such analysis in this study is likely to bias the outcome.

Adverse events were recorded stringently, and this is reflected in a fact that most of the participants experienced an adverse event. However, these were mostly minor or related to the constipation *per se* and there was no mortality associated with the therapy. In about one-third of the patients, device-related adverse events required modification of the system including revisions, replacement or removal of whole or part of the system. The potential need for revisional surgery during the course of therapy should be explained to patients prior to implantation. The clinical and financial impact of this maintenance aspect should be considered and compared to the clinical and financial impact of other treatment alternatives when including SNM in a clinical service set-up.

The role of SNM within the treatment algorithm and the clinical treatment pathway for chronic constipation in comparison with other options, as well as patient selection criteria, is unclear. Recent randomized double-blind crossover studies have shown no difference between active and sham stimulations [17, 19]. In both studies, 30–60% of patients had a positive response during sham stimulation, suggestive of either lasting effects of sensory stimulation beyond washout period between sham and active treatment (2–3 weeks) or high placebo effects of this treatment. In the light of these results from the well-designed randomized trials, it is difficult to recommend sacral neuromodulation as a treatment within a treatment algorithm of constipation.
